# Model Integration in Computational Biology: The Role of Reproducibility, Credibility and Utility

**DOI:** 10.3389/fsysb.2022.822606

**Published:** 2022-03-07

**Authors:** Jonathan Karr, Rahuman S. Malik-Sheriff, James Osborne, Gilberto Gonzalez-Parra, Eric Forgoston, Ruth Bowness, Yaling Liu, Robin Thompson, Winston Garira, Jacob Barhak, John Rice, Marcella Torres, Hana M. Dobrovolny, Tingting Tang, William Waites, James A. Glazier, James R. Faeder, Alexander Kulesza

**Affiliations:** 1 Department of Genetics and Genomic Sciences, Icahn School of Medicine at Mount Sinai, New York, NY, United States; 2 European Molecular Biology Laboratory, European Bioinformatics Institute (EMBL-EBI), Hinxton, Cambridgeshire, United Kingdom; 3 School of Mathematics and Statistics, University of Melbourne, Parkville, VIC, Australia; 4 Mathematics Department, New Mexico Tech, Socorro, NM, United States; 5 Department of Applied Mathematics and Statistics, Montclair State University, Montclair, NJ, United States; 6 Department of Mathematical Sciences, University of Bath, Bath, United Kingdom; 7 Department of Mechanical Engineering and Mechanics, Department of Bioengineering, Lehigh University, Bethlehem, PA, United States; 8 Mathematics Institute and the Zeeman Institute for Systems Biology and Infectious Disease Epidemiology Research, University of Warwick, Coventry, United Kingdom; 9 Department of Mathematics and Applied Mathematics, Modelling Health and Environmental Linkages Research Group, University of Venda, Limpopo, South Africa; 10 Jacob Barhak Analytics, Austin, TX, United States; 11 Independent Retired Working Group Volunteer, Virginia Beach, VA, United States; 12 Department of Mathematics and Computer Science, University of Richmond, Richmond, VA, United States; 13 Department of Physics and Astronomy, Texas Christian University, Fort Worth, TX, United States; 14 Department of Mathematics and Statistics in San Diego State University (SDSU) and SDSU Imperial Valley, Calexico, CA, United States; 15 Centre for Mathematical Modelling of Infectious Diseases, London School of Hygiene and Tropical Medicine, London, United Kingdom; 16 Department of Computer and Information Sciences, University of Strathclyde, Glasgow, Scotland; 17 Biocomplexity Institute, Indiana University, Bloomington, IN, United States; 18 Department of Computational and Systems Biology, University of Pittsburgh, Pittsburgh, PA, United States; 19 Novadiscovery SA, Lyon, France

**Keywords:** simulation, reproducibility, crisis, computational modeling, credibility

## Abstract

During the COVID-19 pandemic, mathematical modeling of disease transmission has become a cornerstone of key state decisions. To advance the state-of-the-art host viral modeling to handle future pandemics, many scientists working on related issues assembled to discuss the topics. These discussions exposed the reproducibility crisis that leads to inability to reuse and integrate models. This document summarizes these discussions, presents difficulties, and mentions existing efforts towards future solutions that will allow future model utility and integration. We argue that without addressing these challenges, scientists will have diminished ability to build, disseminate, and implement high-impact multi-scale modeling that is needed to understand the health crises we face.

## INTRODUCTION–THE PROMISE OF MODELING

Starting with the development of the SIR model in 1927 (Kermack et al., 1927), there has been a long history of population-level epidemiological modeling. The idea of being able to forecast biological phenomena computationally seemed very promising and as a result it has engaged researchers for a long time. Within time, many promises were made in the name of modeling, and enough effort was spent to warrant evaluating current capabilities, concepts, and hurdles.

Over the years, these studies have included the addition of population compartments, as well as the inclusion of age structure, vaccine, and quarantine, to name just a few advancements. Other studies have moved away from classical deterministic models to better understand the role of stochasticity. Moreover, this wide range of epidemic models has been used to study numerous diseases. Because of the vast amount of knowledge that has been gained through epidemic modeling over approximately the past 100 years, it was possible for researchers to quickly adapt their models to make predictions about the spread and control of the SARS-CoV-2 coronavirus.

However, unlike the population-level epidemic modeling effort, much less is known about how viral infections spread throughout the body, including its immune response and the response of different organ systems. Moreover, very little is known about the connection between infection at the individual scale and infection at the population scale.

Some examples like (Azmy et al., 2018) and (Ackleh et al., 2013) use bacteria or virus load as a feature parameter in partial differential equation compartment systems, where the progression of the disease among the population is linked to the virus load in infectious individuals.

Within-host models can explore how the variance of biology within the body can impact both a disease and its treatment (Zarnitsyna et al., 2021). Such mathematical models of viruses focusing on the in-host dynamics should be developed and utilized to a greater extent.

Multi-scale within-host modeling is common, with scales from the molecular and cellular levels integrated successfully with the larger whole-organ or whole-body scales (Powathil et al., 2012; Bowness et al., 2018), (Segovia-Juarez et al., 2004). There are fewer models, however, that successfully combine within-host models with population-level models. One barrier to the development of such models is the potential to lose the within-host granularity that is often seen when integrating to a higher scale. Many researchers may question if these multi-scale models truly offer new insight or if they are more informative when analyzed independently. Such questions emphasize the challenges of multi-scale model integration.

As we move from conceptual models (the diagrams and verbal models of biologists) to mathematical models to computer code, we gain in executability, but we lose shareability. Shareability requires multiple concepts: Reusability–someone else can run the same computer code, perhaps with different initial conditions or parameter values; Extensibility–someone can add modules or replace modules within the model without breaking it; Extractability–someone can select model components and use them, and they continue to function independently of their initial context; Portability–the model can be reused in a different computational instantiation from its original implementation. In particular, the knowledge embedded in computer code is generally stranded or lost, since you cannot easily infer the underlying conceptual model from the mathematical model or the mathematical model from its computer code. As a result, an essential aspect of model development is a formal process that begins with a detailed and complete specification of a fully sharable conceptual model, then develops a less sharable, but quantitative mathematical model, which is an interpretation of the biology and physics of the conceptual model, and finally a computer simulation, which implements the mathematical model in the form of specific algorithms and methodologies. At each step we need to define additional parameters and concepts.

One relatively new key concept enabling construction of models of complex phenomena is composition (Halter et al., 2020). Decomposition lets us break down a complex problem into simpler problems that can be solved or simulated and composition lets us systematically recombine these solutions into a solution of the original problem. To accomplish this, we need to think in terms of higher-order operations on models: what the models are is less important than what can be done with them. There are two kinds of composition: parallel and serial. Parallel composition means running models concurrently. This is useful for ensemble or consensus approaches that combine multiple models to arrive at a best estimate. Serial composition is when the output of one model becomes the input of another. It is important to think about the type of the model, what input it requires and what output it produces because compatibility is required for serial composition. Serial composition has been used to great effect, for example in whole cell models. Of course, it is possible to compose these combined models.

Combination of models can be from white box models or black box models. Black box models do not expose much information to the modeler, but even models that are composed of white-box models may suffer from transparency shortcomings due to the composition. In modular models, three different types of submodel couplings can be found: 1) black-box models with code-level coupling using information-hiding interfaces, 2) white-box models with code-level coupling and 3) white-box models with biological-level coupling. Compositionality and modeling has been extensively studied theoretically and the primitive operations, parallel and serial composition, explored in detail for certain classes of model (Baez and Master, 2020; Baez et al., 2021) that appear in fields as diverse as electronics engineering, chemistry, molecular biology (Danos and Laneve, 2004), plant biology (Honorato-Zimmer et al., 2018), infectious diseases (Halter et al., 2020; Waites et al., 2021) and economic game theory (Atkey et al., 2020). However, to address practical problems across scales, infrastructure is required. First, it is necessary to be able to discover models; models cannot be composed if they are unknown or unavailable. To do this, a catalog is needed with metadata about models and how to obtain them. We note the existence of mature software for data catalogs that is easily repurposed (CKAN, 2021). The models must be described sufficiently well to know if they can be composed, annotated with information about their input and output types. Annotations facilitate auxiliary tasks such as searching for appropriate models and ascertaining provenance. Finally, attention is needed to the detail of composition of a broad class of models, recognizing that errors introduced by (de)composition are only well-understood for some cases (Hairer et al., 2006; Blanes et al., 2008).

With so many multiscale modeling methods that are seemingly disjoint and mutually exclusive, recent efforts have sought to bring some order in the discussion of multiscale models of pandemics by providing a complete categorization of them (Garira, 2017; Garira, 2018). These publications identified five different categories of multiscale models of diseases that use different integration frameworks to integrate across scales. While this categorization cannot be claimed to be unique, it constitutes a good starting point, which may be found useful as a basis for further refinement in the discourse of multiscale modeling of pandemics. The paper (Garira, 2018) further identified ten of the most significant challenges that stand in the way of future advances in integration across scales in the development of multiscale modeling of disease dynamics. Collaborative research among scientists with different skills is needed to fully resolve these challenges.

The recent COVID-19 pandemic has highlighted the importance of modeling the disease and its potential vulnerabilities for interventions. Notable examples include (Russo et al., 2020) and (Russo et al., 2021) that develop models for vaccines. Between-host infection simulations helped researchers to make forecasts about the spread and potential control of the coronavirus. These models build from a century of work in population-level epidemiological modeling. While simple models have been attempting to assess potential antiviral drug combinations, very little is still known about the connection between infection at the individual scale and infection at the population scale (Dodds et al., 2021).

Indeed during the COVID-19 pandemic, many scientists working on related issues assembled under the umbrella of the Multiscale Modeling and Viral Pandemics Working Group (Multiscale Modeling and Viral Pandemics, 2021). This group is part of the MultiScale Modeling (MSM) Consortium hosted by the Interagency Modeling and Analysis Group (IMAG) (Interagency Modeling and Analysis Group, 2021). The discussions about modeling and its promise collected many ideas that were represented there, many times representing more than one opinion, creating a choir of voices. Among those voices, the group located many hurdles that might explain why the promise of modeling is not yet fully fulfilled–those are discussed below and organized considering the workflow of reproducing, gaining credibility, reuse, and integration.

## FROM REPRODUCIBILITY TO CREDIBILITY TO UTILITY TO INTEGRATION

**FIGURE 1 |:**
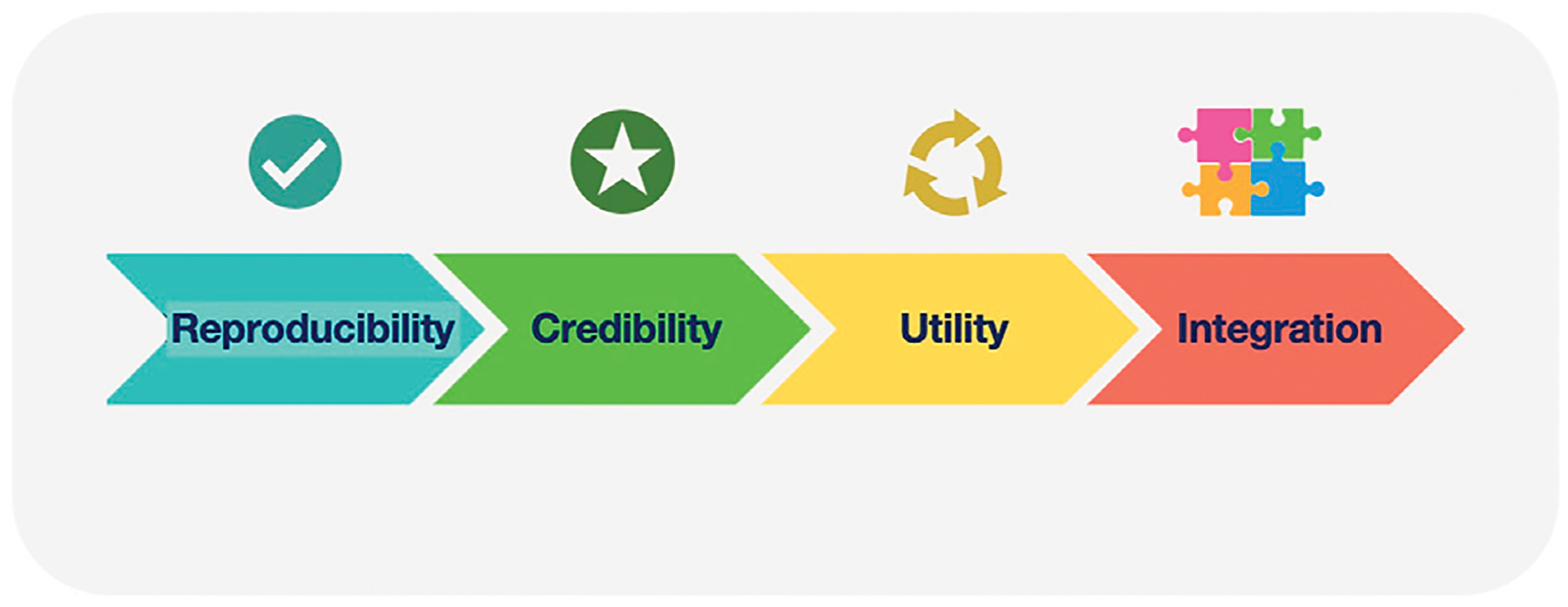
Path towards model integration.

Multi-scale models are intrinsically complex, and usually are modular, whereby the model is divided into units that interact with each other. Modularity facilitates component reuse and model integration, including the ability to exchange modules during, or between, simulations has many advantages (Petersen et al., 2014), but it depends both upon the validity of each individual module, as well as the ability to connect modules, so that they inter-operate appropriately. The promise of multiscale, modular modeling is that researchers can build from each other, using prior published models and building blocks for new, more accurate or more impactful models. For this vision to be achieved, 1) models must be reproducible, so that researchers are assured the module will perform as expected, 2) models must be credible, so that researchers are confident that reusing a module will be useful and appropriate, 3) models must be reusable, meaning not only can they reproduce published results, but also that they can be modified to fit new contexts, and 4) researchers must be able to integrate models with other models. Figure 1 shows these four steps schematically, including how each step depends on its predecessors.

The discussions in this group raised many issues that prevent model integration that start with inability to reproduce models, which leads to low credibility of those models, which reduces reuse, which leads to inability to combine and integrate larger more complex models. Therefore, we address many issues at lower levels that will help reach integration.

### The Reproducibility Crisis

Computational biomedical modeling involves mathematical representation of biological processes to study complex system behavior and was expected to be less affected by the reproducibility crisis. After all, computer software should be deterministic and therefore repeatable if designed well, compared to biological processes that have a random nature where experiments are not guaranteed to repeat themselves and many repetitions are required for the mean to converge.

However, models often fail to be reproducible and the reasons for the failure and prevalence are not fully understood. In a recent study (Tiwari et al., 2021), the BioModels group analyzed 455 kinetic models published in 152 peer-reviewed journals, a collective work of about 1,400 scientists from 49 countries. Most of these models were manually encoded from scratch to assess the reproducibility. Their investigation revealed that 49% of the models could not be reproduced using the information provided in the manuscripts. With further effort, they managed to reproduce an additional 12% either by empirical correction or support from authors. The other 37% remained non-reproducible due to missing parameter values, missing initial concentrations, inconsistent model structure, or missing information. Among the corresponding authors they contacted less than 30% responded. Models from many life science journals failed to be reproducible, revealing a common problem in the peer-review process. The group proposed an 8-point reproducibility scorecard to assess each model and address the reproducibility crisis. A similar study that reports similar deficiencies is reported in (Kirouac et al., 2019). The term crisis is not exaggerated and is well justified since the need to combine models together already exists and building blocks should be solid and match expectations. The “promise of modeling” should be fulfilled already, yet we find modelers consistently unable to reproduce basic steps—thus stagnating instead of innovating, or even worse—backtracking progress. The numbers quoted above cannot be ignored or claimed as being normal—thus the term crisis is used.

This shows only part of the larger crisis. The ideal we would like to reach is repeatability and reproducibility of models in publications and repositories. Repeatability is the ability to repeat the same experiment with the same system, and reproducibility is the ability to repeat the same experiment by another scientist without the same system. The goal here is to have all aspects of the simulation pipeline (Biological Model, Mathematical Model, Numerical Methods, Computational Implementation) be auditable, i.e., they should be fully described (as appropriate for each component) to enable results to be repeated and reproduced. However, the modeling community is far from this ideal. With the diagnosis of this problem, different groups aim for collaborative efforts to set up best practices.

It is important to acknowledge that the situation is much worse for other aspects of modeling. For any other aspect, it would be hard to even conduct such a study to evaluate reproducibility because the information often is not systematically cataloged let alone shared in a common format. Examples include how models were constructed, calibrated, or validated. Compared to the software industry or finite element modeling that have reached well established methods of exchanging information and repositories, such as file exchange formats/git, computational biological modeling has a long way to go and in this paper we will try to address some topics to be dealt with. If we cannot reproduce models, how can those be considered credible by other modelers, stakeholders, or even the public?

### Credibility of Models

In a larger context, model reproducibility is strongly tied to model credibility in light of a given purpose. A model without a purpose is a mere exercise suitable for the classroom and therefore the prerequisite for any realistic application is the design and testing of the model with that specific purpose in mind (often called the “Context of Use”, see below). Often, not only the model developer, but also others will need to confirm or challenge the credibility of a model and thus, a model built for a specific purpose must be at least repeatable and it is highly recommended it be reproducible. In turn, a model that is not repeatable, cannot be reproduced, and therefore cannot be deemed credible by others (who cannot understand the internals, especially if expert modelers cannot reproduce it). Therefore, the proof of model repeatability and reproducibility lies with the modeler who needs to prove the value of the model for a given application.

A modeler should consider the model’s purpose from the start of development and consider: for what; by who; what is the level of knowledge and skill of the user; and in what environment. If only the “for what” is specified, this implies the model can be used by anyone on any system, which broadens the scope and reduces the chance of reproducibility and hence reduces credibility by the potential user.

This proof of value becomes of utmost importance if critical decisions rely on a model. There are, in fact, many cases where human lives depend (directly or indirectly) on a model.

In most cases, users expect a system based on a model to be accredited somehow before use. In analogy, a physician will also not use a medical product, for example a medical device, that is not approved and tested for the anticipated use—because of the risk to harm the patient should the product fail or not work consistently.

This accreditation role many times falls on government agencies such as the FDA, or NASA. Those agencies have different approaches towards credibility of models. Guidelines issued by authorities regulating the use of such models give a “gold standard” recipe for how a modeler can ensure reproducibility and establish credibility of a model.

NASA takes modeling seriously. After the space shuttle Challenger disaster NASA rewrote a standard (NASA, 2016) and wrote guidelines (NASA, 2019). An interesting component in the NASA approach was a risk-adjusted approach that considered both the probability and consequences of a modeled systems failure, in which the level of risk raises or lowers the bar for the data needed to accredit a model. This approach also helps with cost.

FDA regulates the use of medical devices and drugs and also assesses computational models submitted as part of the market authorization dossiers. For many years, simulations have been part of these dossiers. If models can systematically shortcut and prevent issues related with long and costly clinical trials still needs proof, but the number of submissions to the FDA under the use of models has been constantly rising over the past years. The FDA has released and adopted guidance documents on the reporting and validation of computational models for regulatory submissions, (FDA, 2016; FDA, 2003), and actually considered the NASA standards when creating those (FDA, 2016). In 2018 the American Society of Mechanical Engineers (ASME) issued an important guidance ASME V&V 40 (ASME, 2018) of how to assess credibility of computational models of medical devices through verification and validation (V&V). The guideline is centered around the definition of the context of use (CoU) of the model, which is formulated based on the questions of interest the model will answer. The CoU is then analyzed in terms of the “model risk” - being the influence the model exerts on a decision and the potential consequences these decisions might incur. Commensurate with this model risk, the modeler establishes the credibility goals, performs verification validation and uncertainty quantification actions, and then assesses the outcome of this exercise in order to allow judging of the acceptability of the model CoU. Key to this guidance is its overarching nature that also allows adoption in other (e.g., drug development) fields irrespective of the model type (Viceconti et al., 2019; Kuemmel et al., 2020). Very recently, an FDA guidance draft has been updated taking into account this standard (FDA 2021b). In the paper by (Viceconti et al., 2019) the verification, validation and uncertainty quantification (VVUQ) pipeline is streamlined to different types of models. It is, perhaps, the closest to score credibility across model types from mechanistic physics-driven models to machine learning models. However, it is still short of including very recent developments such as ensemble models, although it touches upon the topic.

The FDA understood the potential value of models and modeled data to make developments of medical devices in an efficient manner. Early in the collaboration with the device industry about use of model data in trial processes, the FDA suggested having a “library of “reusable”, “regulatory grade” models. The FDA passed on the idea but is revisiting the library idea given models that meet the information guide for first accreditation. The idea is that the FDA understood the model and it proved useful, so they can accredit it much faster (cheaper) for reuse on a very similar application. Time will tell if this approach works.

In order to advance modeling and simulation fit for regulatory application, FDA and pharmaceutical companies engage in a model-informed drug development (MIDD) pilot program (FDA, 2021a). This pilot program was released in response to a performance goal agreed to under the sixth iteration of the Prescription Drug User Fee Act (PDUFA VI), included as part of the FDA Reauthorization Act of 2017 and advises how particular MIDD approaches can be used in a specific drug development program (Zineh, 2019), and how to report those complying with existing guidelines for regulatory submissions. While for MIDD, commonly data-driven or phenomenological models are used, more complex and multiscale models are coming of age and get submitted to regulators (Viceconti et al., 2021). Data from relevant MSM tools to refine, reduce or even replace trials, could provide additional economic incentives to sponsors (Galluppi et al., 2021). Likewise, however, especially complex models and MSMs models that do not meet the FDA requirements for credibility of model data, will fail to be considered. Guidelines, especially for complex multiscale models are still lacking and thus adoption of other guidelines e.g., ASME V&V40 is needed and discussion with regulatory agencies should be conducted before submission (Musuamba et al., 2021). Competitors focused on the regulatory side will out-compete those that do not, or cannot, comply. It will therefore be necessary to improve available guidance and standardization efforts with regards to repeatability, reproducibility, and reuse so that guidance can be adopted cross-community and entity, by academic and commercial ones alike.

Despite the importance of developing a model with a question of interest and the respective context of use (CoU) in mind, it is important to note that the past paradigm used towards model acceptance/credibility may change in the future. For example there could be multiple motivations for developing a model, motivations could change over time, and someone else could find a new use for a model that was intended for another purpose. Since some contributors to this manuscript have a less strict opinion regarding models being developed with a purpose in mind, we therefore recorded the range of opinions in this manuscript.

An example of a less strict approach to model credibility are new ensemble techniques, such as in (Barhak, 2016; Barhak, 2017), which allow judging a model by its performance in a group of models. This is similar to building teams in sports, where each individual contributes to a team and the value contributed to the team can be determined. Ensemble models allow assigning influence to single models and judging their performance by validation in different scenarios. Thus assigning a score to the model and its assumptions compared to others is possible. So the idea of credibility score may evolve through time and government agencies should consider this newer approach towards credibility. However, even if reproducibility and credibility are amenable there are many issues that prevent reuse of models.

While guidelines and concepts of how to establish credibility of models—even for critical applications—do exist, the field is still evolving and lots of work on completing, harmonizing, and adopting these guidelines still exist. One central question is what a minimal requirement might be for a model to be credibly re-usable.

## ONGOING DIFFICULTIES IMPEDING THE UTILITY AND INTEGRATION OF MODELS.

**FIGURE 2 |:**
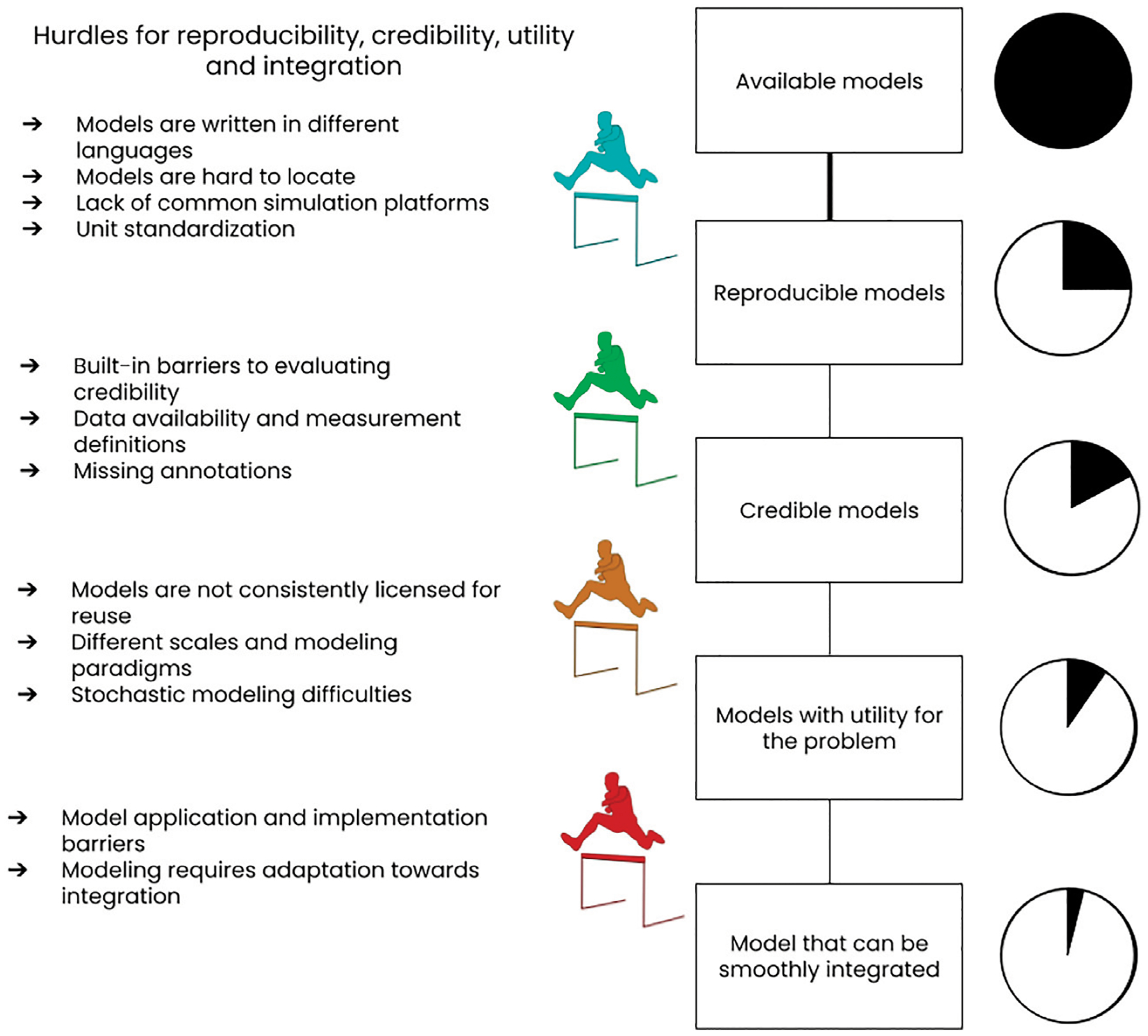
Sketch of difficulties that impede reproducibility, credibility, utility and integration of models, especially in computational biology. An assignment of these difficulties to the four different concepts interpreting them as hurdles is attempted. We would like to point out that the sequential assessment model indicated in the graph is only one of the possibilities a modeler could use to assess the suitability of available models. In the rest of the paper we therefore address each difficulty separately.

Since there is a large variety of known issues that prevent reuse and many solutions, we have divided them by topic. For simplicity Table 1 describes the difficulties and possible solutions:

We also attempted to spread those difficulties as hurdles that relate to reproducibility, credibility, utility, and integration. Figure 2 depicts this analogy.

The paper continues elaborating on those topics and expands explanations hereafter.

### Built-In Barriers for Evaluating Model Credibility

If one considers models that exist currently, what impedes third parties in assessing their credibility according to “gold standard” guidelines (discussed before)? Most of them are not designed, too little or not transparently documented or supported with material allowing a third party for such credibility assessment.

Models include assumptions that need to be specified. Users need to know, under what conditions the model is appropriate? This is a question asked by any modeler. More provenance information is needed for reuse and composition. Another investigator who wants to expand a model may need to know what the assumptions or design decisions were so they know how to appropriately modify or expand a model. A regulatory body might want to be able to trace a model back to the data sources that informed it. Someone who wants to re-train a model for a different cell type or tissue might want to trace the data back to know what aspects of the training data need to be replaced. Enhancing model credibility can be achieved through enhancing documentation, establishing best practices, and tests.

Examples of information suggested to include in documentation are the design decisions that motivated a model, what the model is designed to explain/forecast, and explanations of data sources that contributed to a model. Ideally, this would include links to data repositories, indicate which assumptions were used to interpret the data, consider the methods/tools/users associated with model calibration, and evaluate if the model fulfills its intended purpose (Parker et al., 2002).

In addition, documentation should describe model limitations. It can be difficult to quickly determine which populations or scenarios a model can be reasonably applied to. This information can usually be teased out by carefully considering the data that has been used for fitting or validation, as well as digging through the discussion or conclusion. However, some doubt often remains because of the natural tendency to promote one’s work, and the, perhaps unrealistic, expectation that publishable work be as widely applicable as possible. If it were standard practice in model reporting to recommend specific model applications, this could provide clarity for those implementing or extending the model.

Beyond design and implementation, best practices should include reports of tests that describe what was simulated and the experimental or other data that was used to evaluate the test. Unlike software test reports which focus on failures, these reports must also focus on passes because they help establish the domain under which the model has been established to make trustworthy predictions. For example, this establishes the domain under which their clinical use would be supported. When considering test implementation, some suggestions emphasize the need for a structured approach with unit-test style tests (Sarma et al., 2016; Gerkin et al., 2019) (Lieven et al., 2020) and continuous evaluation of such tests similar to continuous integration of software (Meyer, 2014; Krafczyk et al., 2019; Zhao et al., 2017). To enhance model credibility further, the model description should include validation tests against independent data, uncertainty assessments, and peer reviews (Refsgaard et al., 2005; Jakeman et al., 2006).

### Models Are Written in Different Languages

When modelers do use a consistent, declarative language to describe their models, these models can then be stored and searched in readily-available repositories. The BioModels collection is a good example of such a repository for Systems Biology Markup Language (SBML) (Systems Biology Markup Language, 2021) models. As another example, the Physiome Model Repository (PMR) is a collection of CellML models. Although these repositories are a good step forward toward finding and reusing published models, by themselves, they are insufficient.

First, there are often significant differences between modeling languages–e.g., the CellML language and SBML are almost opposite in their approach to capturing the information in a model. Second, even within one modeling language it can be difficult for an outside user to understand the biological and mathematical content of a model written by someone else. As with software engineering, the key to enabling understandability and reuse of models is to provide unambiguous documentation about the intended semantics of the model.

One major problem we face for many kinds of models, which SBML and SBGN (Kitano, 2021) and projects like Biotapestry address partially for biological networks, is that we lack tools and formalism for consistently building, annotating, representing, displaying and manipulating conceptual models of complex biological phenomena with a spatial component. We lack standards for all of the key elements that need to be represented: the objects, the processes (behaviors and interactions) they participate in, the initial and boundary conditions and the dynamics and events that govern their evolution.

In many cases we also lack the scientific understanding of how to convert these conceptual models into mathematical models because we lack the “constitutive relations” that are the equivalent of the standard rate laws for chemical reactions. In this case we don’t have an agreed upon way to parametrize the submodels and to define their inputs and outputs.

Another big missing piece is a language to describe the possible experimental manipulations or perturbations of a biological system. We have concentrated on building mathematical and computational descriptions of biology, but not on the things we can do to them. Without such a description, classical techniques like perturbation and sensitivity analysis are much less useful. If we want to achieve a desired outcome by manipulating a given biological system, we need to know the constraints in our ability to manipulate that system. Knowing that we could achieve what we want by increasing the value of k_xx by 25% is not actionable unless we can increase k_xx. The lack of orthogonality in biology (any perturbation of a biological system affects many aspects simultaneously, is what makes mathematical models so valuable for understanding (we have clean control parameters). But it also reduces their utility in designing experiments or clinical interventions. We need models that combine the model of the biological system with a model of the space of possible experiments. The sensitivity of this combined system is what tells us what is achievable in the lab or clinic.

Understanding the biological content of a model is critical to both reuse and reproducibility. If the model itself is incomprehensible, how can one know what its expected behavior and performance should be under different conditions? Semantic annotation is not necessary for simple repeatability, but if our goals include reproducibility and reusability, then we must make explicit and clear the biology and physics that underlie the model. An ideal modeling language should address this, yet until such a standard language is established we are faced with a need to integrate among different languages.

One simple integration example (Another example of integration and reproduction of a model, 2021) involving two popular languages, python and MATLAB, demonstrates the problem of transition between languages. There is no real translation between languages. No general compiler exists between multiple languages and human efforts are required. Fortunately, there are standardization efforts among languages.

The standardization problem is not new and was considered by modelers a long time ago, resulting in the Systems Biology Markup Language (SBML) (Systems Biology Markup Language, 2021) that is a very helpful format that can help transport models between systems. SBML has a track record of success and allows transporting models between hundreds of systems. However, despite its popularity, it is not an official standard and the community decided not to go in that direction (Recent SDO/COMBINE legal entity issues, 2021). Note that there are many similar community standardization efforts aggregated in the biosimulation modeling community known as COMBINE (Computational Modeling in Biology Network) (COMBINE, Online). COMBINE includes SBML as well as many other specifications, yet those communities are still in the process of standardization and need to organize legally.

Nevertheless, the lack of legal governance does not stop communities from developing even more tools for result handling and analysis like PETab (Schmiester et al., 2021), SED-ML (Waltemath et al., 2011), SESSL (Ewald and Uhrmacher, 2014), KiSAO (Courtot et al., 2011), SBRML (Dada et al., 2010), HDF5 (Folk et al., 2011), Vega (Satyanarayan et al., 2014; Satyanarayan et al., 2017), ggplot2 (Wickham, 2011), and others. Those tools show actual needs by the community, but these are much less mature and much less adopted. Their capabilities need to be expanded; they need to be adopted; software tools need to support them; and there needs to be infrastructure to share them, such as a repository. Another piece is that the software tools needed for the above are scattered, plus it is often unclear what subset of the above they support, and tools often become inaccessible. Tools need to be submitted to registries and the capabilities need to be annotated.

There is a need to coordinate the various standardization efforts that are needed for the different scales and biology that need to be involved in multi-scale models. The need for multiple standards may be recognized, yet the need to coordinate them to be able to compose multiscale models has received less attention.

### Models Are Hard to Locate

Many times model location is a difficult task since models are published in different sources. Despite many repositories available there are many ways models are published including journal papers, conferences, preprint services such as BioArxiv, web sites, and code repositories such as GitHub. In some good cases, there are model archive/linking web sites such as BioModels (BioModels, 2021), SimTK (SimTK, 2021), IMAGWiki (IMAGWiki, 2021), and in the future modeleXchange (Malik-Sheriff et al., 2020). However, currently there is no one aggregator that helps locate all models and many times community members cannot agree on location and attempt to create more repositories rather than centralize efforts.

Moreover, simulation workflows are even harder to find. For example, BioModels primarily focuses on models. There has been much less focus on publishing the construction/calibration of models, simulations, their results, analyses of their results, or entire workflows for the above. Sharing all of this needs embracing other repositories and developing some new ones.

We recommend that modelers use those repositories since we are at a stage in evolution of modeling where model composition is of interest and availability of modeling components is important. We ask that modelers consider from development to start permissive Intellectual Property for the versions published in those repositories to increase accessibility.

### Lack of Common Platforms for Executing Models and Simulations

Even if models can be located, their simulation is a different issue. Due to the existence of many partially supported standardization efforts in this field, it is often difficult to know what tool needs to be used with which model. It can also be difficult to find that tool, download it, install it, learn it, and to use it, especially for large simulations. These issues keep modelers in silos.

Even formats associated with standardization efforts have difficulties. It is not possible, for example, to load a MATLAB/SimBiology SBML L2V4 model into COPASI and someone adhering to latest standards implementing L3V2 SBML support for import will find difficulties in importing models. Moreover, it is difficult to find a common platform that supports all SBML versions. This version compatibility gap is not uncommon. However, since biological models take a long time to develop and represent phenomena that will persist for long terms, it is important to have long term stability and support with newer platforms and older models.

If the goal is for non-modelers to be able to interact with models (e.g., to analyze data, to contribute data toward a modeling project, or to apply a model for medicine), it needs to be much easier to find and use these tools. Two initiatives that are trying to address this are BioSimulators (BioSimulators, 2021) and runBioSimulations (Shaikh et al., 2021).

### Modeling Requires Adaptation Towards Integration

Many times the models as published need some level of manipulation to plug into another model. For example in (Castiglione et al., 2021) the survival function needs adaptation to transform it as can be seen from the public discussion in (About using a multi-scale mortality model in the ensemble, Online). Note that all those models need to be scaled to the same units and scales. Another example is in (Ke et al., 2020) where infectiousness is proportional to max infectiousness while the models in (Hart et al., 2021) are density models. In the model in (Castiglione et al., 2021) the time scale was originally 8 h and it needed to change to daily probability to merge into another model in (COVID19Models/COVID19_Mortality_Castiglione at main, 2021), which required scaling of the probability function. Those examples are relatively simple integrations and in more complex integrations the adaptation effort is more significant and many more obstacles exist.

One obstacle is lack of standards for describing composite models and software tools for merging models. One specification is SBML-comp, but it is cumbersome and few tools support it. Another tool is SemGen (Neal et al., 2019a), but it focuses on finding mappings between similar models. To the point here, SBML-comp is designed to compose models that were not intended to be composed. Instead, composition needs to be deeply ingrained into the entire community so that models are anticipating the needs of composition from the beginning.

Note that adaptation towards standardization also requires matching terminology, and especially matching of units of measure, as well as proper documentation which we will address in the next topics.

### Unit Standardization

Unfortunately, units of measure are not yet standardized, an open problem despite many attempts to resolve it by multiple standardization bodies such as IEEE, CDCIC, and NIST. One indication of the severity of the problem is that a Github search for “unit conversion” shows over a thousand results. Another good example of the severity of the problem is ClincialTrials.Gov that aggregates quantitative data from around the world and this database shows over 24 K different units of measure (Barhak and Schertz, 2019). One attempt at solving this standardization issue using machine learning is ClinicalUnitMapping.com, yet this project requires more effort.

Unit mismatches become particularly problematic when trying to integrate models across different spatial or time scales. For example, intracellular processes occur on micrometer spatial scales and seconds to minutes time scales. An in-host, tissue-level model of infection processes operates at millimeter to centimeter distances and hour to day time scales. When trying to integrate the two into a single multiscale model, care must be taken to ensure appropriate conversion of units when transferring output of one model as input to the other. This broad range of spatial and temporal scales can also cause computational problems requiring development of new algorithms to make computation more efficient across multiple scales (Jung and Sugita, 2017). While it is impossible to avoid having to convert units, we are advocating for clarity in the use of units. Sometimes models are used (and published) without specifying the units used for simulation parameters—this is a practice that needs to be corrected. Moreover, tools such as ClinicalUnitMapping.com can help overcome standardization difficulties in the future with more development. Once mapping to standardized units is easier, then simulation parameters can be converted appropriately when different scales or units are needed.

Lack of standard units for measurement of infectious virions is particularly problematic when trying to develop stochastic viral models. Stochastic models often require that we track individual infectious viral particles, yet it is not clear how the typical viral titer units of TCID_50_/ml and pfu/ml convert to individual virions. Two attempts have been made to estimate the conversion factor, both for influenza, resulting in estimates of 1 TCID_50_/ml of nasal wash corresponding to 10^2^–10^5^ (Handel et al., 2007) or 3 × 10^4^-3 × 10^5^ (Perelson et al., 2012) virions at the site of infection. Such order of magnitude uncertainty in unit conversion makes it difficult to develop accurate model representations of viral infections. We are not advocating for a standard conversion factor, since the conversion factor likely depends not only on the specific virus, but also conditions such as temperature and pH, which are known to affect viral infectivity (Rowell and Dobrovolny, 2020; Heumann et al., 2021). Rather, we are advocating for development of new viral measurement techniques that can more reliably quantify the number of infectious viruses present in a sample.

### Data Availability and Measurement Definitions

When attempting to integrate models, the phenomenon being reproduced by the models or the data they are based on might not be the same. This is especially true when model definitions evolve or can change in many ways. Examples include International Classification of disease (ICD) codes (Wikipedia, 2021a) that went through multiple versions through the years, or even a disease definition that has evolved for sepsis (Gary et al., 2021). Even outcomes of clinical trials change if counted using different definitions as seen in (clinicaltrials.gov/NCT00379769 - GlaxoSmithKline, 2017). Those definitions can hinder connecting different models together. Possible solutions are machine learning techniques that can transfer interpretation or modeling techniques that merge human interpretation from multiple experts into the modeling process (Barhak, 2020a).

Another issue specific to models dealing with viruses may seem like the lack of unit standardization for measurement of virus. However, it is a measurement definition issue. Infectious virus concentrations are measured using TCID_50_/ml (50% tissue culture infectious dose) or in pfu/ml (plaque forming units), both of which depend on specific experimental conditions such as temperature, humidity, and measurement time. Studies have shown that even a lab using identical experimental conditions cannot reproduce the same measured experimental values of virus leading to differences in estimated parameters for models (Paradis et al., 2015). There is also an underlying assumption for both units of measurement that an observed plaque was initiated by a single infectious virion, which has never been clearly proven to be true. More recently, non-infectious virus particle concentrations are being measured using PCR. In this technique, the number of segments of a particular piece of RNA are measured. While this unit is more tangible and consistent than the infectious viral titer units, viral kinetics models often consider only infectious virions. Although non-infectious viruses are starting to be incorporated into models, the relationship between infectious and non-infectious virions changes over the course of an infection (Petrie et al., 2013), making it difficult to use these measurements to get at the underlying infectious virus dynamics. New measurement techniques and strategies for more direct measurement of infectious virions are being developed (Cresta et al., 2021).

Data availability to rationalize calibration and validation of models is crucial but often not possible because of data sharing policy and privacy (especially for individual human data). Moreover, undisclosed data from industry sponsored clinical trials used in model building and validation generally excludes many useful models from any assessment by the scientific community. This is a difficult problem but there may exist some partial solutions. Synthetic data that is statistically similar to real-world data without containing information about any real individual can be shared. While the similarity can only be evaluated with access to the original data as in (Stack et al., 2013), because it can be shared, it can be used for calibration and validation of other models (Barhak, 2017; Ajelle et al., 2018; Liu et al., 2018; Wang et al., 2019). There is a risk of error using synthetic data in this way since, though it may have been similar in some respects to the original data, it might be different in some other respect that matters for a model different from that used for validation. For validation of model results against individual data that cannot be shared, it is conceivable that services could be deployed to query the data. Differential privacy (Dwork 2008; Garfinkel and Leclerc, 2020) to establish a privacy budget for such a service providing an information theoretic bound on how much information is allowed to be revealed in response to queries. This budget can be set to whatever is considered ethically and administratively acceptable. More research is needed to adapt this idea to suit model making needs.

Even data that are publicly available has limitations. In the case of within-host viral kinetics models, sampling of viral loads and immune responses is often not done frequently enough or long enough to ensure parameter identifiability (Miao et al., 2011). During the recent SARS-CoV-2 pandemic, several attempts were made to parameterize within-host viral kinetics models using viral loads measured from patients, but these measurements were often collected only after a patient was hospitalized, so the crucial viral growth phase is missing (Hernandez-Vargas and Velasco-Hernandez., 2020; Wang et al., 2020). Additionally, viral loads were measured via nasal swab, though it is not clear that the viral load in the nose is correlated to the viral load in the respiratory tract, which is the infection location simulated in the models. Other viruses can also infect internal organs that are difficult to access for frequent measurements without invasive procedures. Immune responses are often measured using levels in the blood, which is typically not the site of infection and often not the location of viral load measurements either. Since models are attempting to replicate virus and immune dynamics at the site of infection, these data collection limitations make it difficult to collect the data needed to accurately parameterize such models for humans.

A further methodological issue with some of the available *in vitro* and *in vivo* experimental data is that it often does not represent the infection time course in a single individual or single experimental well. Clinical trial data is often presented as medians or means taken over all patients. A recent study of influenza infections showed that parameter estimates based on fits of models to a single median viral titer curve do not match estimated parameter values based on fits to individual patients (Hooker and Ganusov, 2021). This also masks patient-to-patient variability in infection. Pre-clinical animal studies and *in vitro* studies can be even worse as animals are often sacrificed and infections in individual wells are stopped to make measurements at each time point. In this case, experimental data consist of an average of measurements from several animals/wells at each time point that differ from the set of animals/wells at other time points.

### Missing Annotations in Models

In biosimulation models, documentation about the intended semantics of the model is captured by annotations - additional information that describes the model, and the biological entities included in that model. Further, these annotations can leverage the rich resources of bio-ontologies–consistent nomenclatures and terminologies that describe the biological world in great detail.

COMBINE has recognized these challenges for understandability and reuse of models and is working hard to disseminate best practices around semantic annotation. COMBINE consensus around annotation is described in (Neal et al., 2019b). This paper describes some key tenants for improved semantic annotation: First, these annotations should be written using a standard format, and one that is independent of modeling languages. Thus, COMBINE recommends RDF as a simple triple-based representation to connect model elements to annotations and knowledge resources (e.g., ontologies). Next, COMBINE recommends that annotations should be stored externally from the source code of the model. Obviously, the annotations should be linked to elements within the model source code, but in order to be language independent, they should be stored separately. Finally, COMBINE recommends that modelers and model building communities provide policies and rationale for choosing which knowledge resources to use for which types of annotations. Otherwise, the same biological entity may look different if different modelers annotate the entity against different bioontologies.

Annotations can be useful for multiple tasks such as: annotation of semantic meaning where biology or real-world relevance is explained, annotation of provenance where the origins of the model and its creators are referenced, and annotation of verification indicating tests the model should undertake and pass. However, there is a lack of sufficient annotation about the components of models. This is particularly because modelers choose not to provide annotation and because tools for describing the semantic meaning of components are just starting to emerge. For example, for biochemical models there’s HELM and BpForms. The lack of such annotation makes it hard to determine the points of overlap between models. In addition, there is a lack of annotation about data sources and assumptions which makes it hard to determine whether models are compatible or what needs to be done to make them compatible. For example, do two models represent the same cell type, tissue, or gender? Hopefully policies will be adopted to resolve this issue.

### Models Are Not Consistently Licensed in an Easy Way That Allows Reuse

Different institutions have different approaches towards licensing, as can be seen from this discussion (Licensing issues, 2021). Therefore, model creators may not be aware of the implications of licensing many times when they publish their models. Moreover, some licenses are incompatible with each other or other forms of Intellectual Property (IP) such as patents (Wikipedia, 2021b). Even open source licenses are quite restricted since they are based on copyright laws, which give the owner rights to restrict usage (Barhak, 2020b). In this sense open source licenses resemble patents and in some cases are more restrictive since patents become public domain quicker. Moreover, community members take different sides with regards to licensing issues as can be seen in this discussion (Issues with regard to Call for transparency of COVID-19 models, 2021). Specifically, one license that will make reuse much easier is Creative Commons Zero (CC0) (CC0 Creative Commons, 2021). This license uses the term “No rights reserved” and makes it easier for models and text to be reused with less restrictions. In fact model repositories such as BioModels require releasing the models uploaded there under CC0 (Malik-Sheriff et al., 2020). However, CC0 license has not been adopted by some (Wikipedia, 2021c). To eliminate the licensing problem, modeling communities will have to abandon old school open source licenses that are based on copyright and create conflicts and recommend releasing models to the public domain using licenses such as CC0.

We therefore recommend that models and their associated data should be published under permissive terms. For maximizing reproducibility and integration, we suggest that the most permissive license possible should be chosen. In that regard the CC0 license would be a good choice, effectively waiving interests of the creator in their works and therefore emulating the public domain in jurisdictions where this is necessary.

### Different Scales and Modeling Paradigms

Models are operating on different spatial scales (population or individual) with different modeling paradigms (continuous vs discrete). A tissue could be modeled as a continuum leading to Partial Differential Equations (PDEs) or as a collection of individual interacting cells leading to an agent-based model. Specification of these two models would probably require different languages.

The fact that models capture different scales or that they don’t consistently capture any single scale creates challenges for composition. One opinion is that the challenge is that the scale of a model is not clearly annotated. To compose models, this forces the composing investigator to try to figure out the scales of each model and how to mesh them. Typically this is combined with lack of annotation of units. When developing a standard for specifying models, developers will probably need standards specific for each modelling approach. One possibility is that a family of specification standards may be created. The need for different formats for different domains and scales will probably create the need for a central place where, especially non-modelers, can find information about these various future standards and which tools support them. Ideally, there would also be a central place where these tools can be obtained and executed so that even non-modelers can easily explore models without having to figure out what software is needed, install it, etc. However, it is still unclear what common practices might facilitate composition across scales and how the various component standards should be architected to facilitate integration.

### Model Application and Implementation Barriers

Models are difficult to be used by a community or government. Scientific, regulation, and social communities have different sets of models and different understanding and standards in models. It is hard to convince and establish a common popular model widely acceptable by a wide range of communities and even adopted by the government. The long term validation and approval process may delay the cycle from model application to implementation.

Many models can increase their utility to the scientific community if their applicability and implementation is easy. These aspects can be improved by means of several features. One crucial feature is the reproducibility of the model since usually this is necessary before the model can be applied by the scientific community. The likelihood that a model is applied to different problems increases if their results are reproducible. The reproducibility is difficult to test if there are implementation barriers. Some of these barriers have been pointed out in this article. Thus, we can infer that implementation issues of the models affect the reproducibility of the models and therefore a broad utility of the models to the scientific community.

Currently many models are difficult to implement and therefore unable to make a real impact. Many of the existing models that are used by decision makers are used because those were implementable. More sophisticated models are many times not used due to a need for proper tools or proper expertise. Therefore, many good ideas remain unused due to implementation difficulties. The solution to this problem is long term and requires education of developers, users, and the public.

### Stochastic Modeling Difficulties

Biological systems are exceptionally complex, involving a multitude of interactions among a large number of components at different spatial and temporal scales. Over the years, much work has been performed wherein deterministic models have been developed to understand dynamics from the cellular level to the population level (Murray, 2002). Although these works have provided much insight, it is known that the mean-field dynamics of these deterministic models do not always capture important phenomena (Forgoston and Moore, 2018). For example, disease population models often have a stable endemic state for reproduction numbers greater than one, and therefore it is not possible for the disease to go extinct in the models. This is in direct contrast to the local extinctions of disease that occur all the time in the real-world (Assaf and Meerson, 2010; Bauver et al., 2016), (Doering et al., 2005; Dykman et al., 2008; Ovaskainen, and Meerson, 2010; Forgoston et al., 2011; Schwartz et al., 2011; Nieddu et al., 2017; Billings and Forgoston, 2018). Similarly, at the within-host level, new infections may or may not establish. This phenomenon can be captured by stochastic models, but is not realised by a deterministic model with a single set of model parameters. Furthermore, deterministic models do not account for the random interactions of cells or individuals, nor do they account for the changes in the model’s rates which are related to random events.

To properly model real-world multiscale dynamics, it is often necessary to use stochastic approaches that allow one to make quantitative, statistical predictions, while simultaneously providing qualitative descriptions of system dynamics. The ability to generate stochastic simulations that provide quantitative statistics for the emergence of new dynamics is increasing with advances in computational power. However, the inclusion of stochasticity leads to a variety of issues related to reproducibility. One concern is associated with noise-induced transitions (Assaf and Meerson, 2010; Forgoston and Moore, 2018) or stochastic resonance in which the deterministic system is qualitatively different from the stochastic system. The output of a stochastic model is a distribution or time-series of distributions. In particular, it is possible to have different outcomes for the same model or set of parameter values (e.g., a multimodal equilibrium distribution). In this case, one must use more sophisticated techniques such as Kullback-Leibler divergence or Wasserstein’s distance to make quantitative comparisons with data or other models. Moreover, while it may be possible to compare distributions generated by different stochastic models, it is often not possible to generate identical, individual realisations.

Stochastic models are critically important. Indeed, unlike deterministic models, stochastic models give rise to probabilistic predictions based on ensembles of realisations. However, in general, these types of models present difficulties that include: 1) how one validates a stochastic simulation; and 2) how one can ensure the repeatability of a stochastic simulation. It is worth noting that the latter issue becomes more problematic when software libraries that support modern high-performance computation (including standard parallel computation as well as GPU computation meant to accelerate simulation), cannot guarantee deterministic reproducibility (DOC, 2021). Potential solutions include the development of tools that guarantee repeatability such as ways to set and record pseudo-random number generator seeds as used by the MIcro Simulation Tool (MIST) (Barhak, 2013) and developing standards to address stochastic simulations.

### Open Discussion

During the work on the paper, a few other items were raised that were not resolved and we assembled those here so those can be addressed in a future version. Some of those issues are visible, yet out of reach, at least for this group of authors and the group invites correspondence on resolving the issues we list below.

How existing Multi-Scale frameworks could be made more transparent with respect to the models they encode and potentially more interoperable. How are most multiscale models encoded? Do they use general modeling frameworks that have been developed for this purpose or do they rely on custom-built code? What general purpose multiscale frameworks exist and what types of model integration and linking do they support? Are there opportunities to develop standards that would make such models more reproducible and understandable? What intermediate steps might be possible? For example, some of these frameworks support integration of SBML models (or models encoded in other somewhat standardized languages). Is it beneficial to leverage existing standardization efforts and to develop new ones for the description and implementation of MSM’s going forward? Right now, it is a bit of a wild west where these models are being developed with submodels as generic pieces of code in general purpose languages like C++ and *Python* that rely only on unstructured comments for documentation.

It is still unclear how various component standards should be architected to facilitate integration. Many of the solutions discussed will require dedicated tools and standards and there will be many of those. It is unclear how distributed and decentralized components will be governed. A resource such as modeleXchange (Honorato-Zimmer et al., 2018) may help investigators navigate this landscape for models, and similar tools may be needed to navigate tools and standards.

There is no common methodology on how to deal with the gap between model parameters, data collection, and standards. This topic should be discussed in the future in light of a potential solution of standardized model development explanation. However, standards have to evolve to handle this issue in proper standard organizations.

The COMBINE (COMBINE - Coordinating standards for modeling in biology, 2021) organization has been helpful to start the standardization process. However, it is not a Standards Development Organization (SDO) and its members rejected joining SISO, which was an established SDO. Therefore the products of this work may not be widely accepted unless the organization matures and adopts a legal entity standing with all regulations involved. However, will the community behind this organization mature enough to adopt legal bindings and regulations?

Common formats for results and visualizations still have not been established despite importance. When models produce results during simulation those should be archived and visualized somehow to help user interactions. A common format to represent results and how to generate graphics to represent will help with credibility and integration efforts.

For models to mature, there is a need to establish testing paradigms. Testing included all general software development good testing practices such as verification of calculation code, regression testing, plus model validation and usability testing. One of the authors recommended that a future model testing practices paper be developed. Another voice mentioned that the ensemble modeling approach includes tests within it. Yet the group reached no conclusion.

Barriers preventing validation reduce credibility and therefore have a negative impact on model credibility, which prevents reuse. One barrier is model validation at different scales: Models at different scales from molecular to population scale are usually validated at different standard and testing samples. Cross-scale validation is very difficult since there are multiple factors involved that influence the outcome of different scales. Another barrier is model validation for practical use cases: Real world prediction from the developed model is challenging because of the complexity of the pathogen spreading process. The real spreading process always has a lot of random social and physiological variables that are hard to be included in any model. With more advanced models and availability of more data, practical forecasts will get more accurate.

Another, difficult topic is spatial models. It seems we are pretty much at the beginning in terms of defining spatial models. We don’t have a quantitative language to specify cell shapes, cell behaviors, or tissue architecture. In many cases we don’t even have a qualitative language to do this.

Bioscience modelers are not alone dealing with utility and reuse related issues. A 2016 report on complex systems engineering challenges (Fujimoto et al., 2017) identified other non-technical barriers in the form of social, behavioral and programmatic barriers that were not addressed among the technical issues in this paper.

These and many other topics may be issues for the group to discuss in the future and readers are welcome to join the discussion.

## CONCLUSION

This manuscript discussed the reproducibility crisis in biological computational models. Many issues and difficulties and barriers have been presented. Nevertheless, some efforts towards solutions already are in progress and have been mentioned. We can categorize those challenges to scientific problems, and cultural and community based challenges.

Examples of scientific problems include the need to build good model integration environments, and to establish how to integrate models across paradigms. We also need to resolve many of the stochastic modeling challenges.

Examples of cultural and community barriers include education towards standardization of units, education towards proper annotation of models. Good tools may help with education by helping do those tasks semi automatically.

It is expected that many solutions will have both scientific and cultural aspects. The list of issues should not discourage modelers from developing models. Instead modelers should view this list as a reference of issues to be solved in the future and issues to avoid. The first step in solving the problem is admitting it exists. With this paper the authors recognize the challenges and admit the current state of modeling needs fixing. Hopefully fixing those issues, starting with reproducibility, will increase model credibility and will facilitate reuse and later integration of models. The long term goal of this group is improving models to achieve better human and machine comprehension of biological processes.

## Figures and Tables

**TABLE 1 |:** Difficulties and possible solutions towards improving model utility.

Difficulty	Potential solutions
Models have built-in barriers to evaluate model credibility	Better modeling practices, documentation, and tests
Models are written in different languages	Common transport specifications such as SBML or CellML, and proper documentation and annotation
Models are hard to locate	Archive web sites such as: BioModels, SimTK, IMAGWiki, and the future modeleXchange
Lack of common platforms for executing models and simulations	Platforms such as BioSimulators, and runBioSimulations
Modeling requires adaptation towards integration	Tools for composing models such as SBML-Comp, and SemGen
Unit standardization	Standardization efforts, and machine learning solutions such as ClinicalUnitMapping.com
Data availability and measurement definitions	Models that merge human interpretation, and newer measurement devices
Missing annotations in models	Adoption of policies such as those COMBINE suggests
Models are not consistently licensed to allow for reuse	Abandoning some old school open source licenses and promoting licenses that release to public domain
Different scales and modeling paradigms	Standardization effort and centralization tools
Model application and implementation barriers	Education of modelers, users, and the public
Stochastic modeling difficulties	Development of tools that guarantee repeatability and standards to address stochastic simulations

## APPENDIX–TRACEABILITY

Draft versions of this paper are available online at: https://docs.google.com/document/d/1cqwXAjBWEiJZ1tUBnf66QVHdHd2fKq_W0py7t4PNVLo/edit?usp=sharing

https://docs.google.com/document/d/1voUSrSpv3AZlC1T-BLa3W4wzHQ5vEdJCVrBbwMUTDiQ/edit?usp=sharing

https://docs.google.com/document/d/1IMEgmdNkx-EsnOjGuegpenSIMmKIkK00Lc8Gred3QxM/edit?usp=sharing

https://docs.google.com/document/d/1Ag4ipuybjtthxgV0YjXqYP7AwwNSYcWh/edit

https://docs.google.com/document/d/1VvyP3YZQdQYjj8DFKOpQ4pn_0pdDGgiT/edit.

https://drive.google.com/file/d/1U_lTHrV6STXWNT3GiCepvsLk1WdYgzN5/view.

Many Email discussions that constructed this manuscript are made public and can be accessed through:

https://lists.simtk.org/pipermail/vp-integration-subgroup/

https://lists.simtk.org/pipermail/vp-reproduce-subgroup/
